# Interleukin‐17, Interleukin‐18, T Regulatory Cells, and T‐Helper 17 (Th17) Cells Play a Role in Heart Failure: A Systematic Review and Meta‐Analysis

**DOI:** 10.1155/crp/5979003

**Published:** 2026-05-24

**Authors:** Sohat Sharma, Jayant Seth, Cameron J. Leong, Simon W. Rabkin

**Affiliations:** ^1^ University of British Columbia, Vancouver, British Columbia, Canada, ubc.ca; ^2^ Department of Medicine, Division of Cardiology, Vancouver, British Columbia, Canada

**Keywords:** chronic heart failure, heart failure, IL17, Th1/2, Th17

## Abstract

**Background:**

The objective of this study was to assess whether interleukin‐17 or 18 (IL‐17 or IL‐18), regulatory T cells (Tregs), and T helper17 (Th17) serum concentrations were different in chronic heart failure not associated with myocarditis compared to healthy individuals.

**Methods:**

We searched MEDLINE and Embase for studies with data on IL‐17 or IL‐18 serum concentrations in patients with chronic heart failure. Forest plots were used to quantify results and depict the standard difference of means, 95% confidence interval (CI), and *p* value. Continuous outcomes were assessed as weighted mean differences (WMD) with their 95% CI.

**Results:**

IL‐17 was significantly (*p* < 0.05) higher in patients with heart failure. The combined effect size under the random effects model (MD = 40.00 pg/mL, 95% CI [6.04, 73.96], *p* < 0.001) showed a significant overall increase in IL‐17 serum levels in heart failure. Th17 was significantly (*p* < 0.05) higher in patients with heart failure, and Tregs are significantly lower compared to persons without heart failure. The random effects model indicates an MD of 1.59 pg/mL (95% CI [0.88, 2.30]), highlighting significant elevation of TH17 levels in heart failure. For Tregs, the random effects model presented an MD of −2.96 pg/mL (95% CI [−4.52, −1.40]), both indicating a significant decrease in Treg levels in HF. Additionally, serum IL‐17 concentrations correlated with the severity of the reduction in LV ejection fraction. For IL‐18, all (five) studies reported a statistically significant increase in IL‐18 levels in HF. The pooled mean difference was 251.59 pg/mL (95% CI: 177.24–325.93 pg/mL) with a random effects model.

**Conclusion:**

Serum IL‐17, IL‐18 Th17, and Treg count have utility for risk stratification for patients with heart failure, as biomarkers for heart failure severity and potential target pathways for treatment.

## 1. Introduction

Chronic heart failure (HF) is associated with substantial morbidity and mortality despite known modern therapeutic advances [1]. Inflammation has emerged as a central contributing mechanism in HF pathophysiology, with elevated circulating proinflammatory cytokines reported in both ischemic and nonischemic HF [2]. Histological studies have further demonstrated myocardial infiltration by activated immune cells, including T‐cells [3] and monocyte‐derived macrophages [4]. The CANTOS trial further reinforced this paradigm, showing reductions in HF‐related hospitalizations and mortality from treatment with the interleukin‐1β inhibitor canakinumab [5].

Among immune‐mediated pathways, T‐helper 17(Th17) cells and their signature cytokine interleukin‐17 have attracted interest. Th17‐related chemokines are upregulated in HF [6]. Th17‐related chemokines are upregulated in HF [7], and experimental models suggest that IL‐17 promotes myocardial fibrosis, collagen turnover, and cardiomyocyte apoptosis [8]. Conversely, T‐regulatory cells (Tregs), which suppress Th17‐driven inflammation, appear reduced in HF and correlate with LV systolic function and N‐terminal pro B‐type natriuretic peptide (NT‐PRO‐BNP) [9, 10]. Elevated NT‐proBNP reflects increased myocardial wall stress and ventricular dysfunction, emphasizing the clinical relevance of immune dysregulation in worsening HF severity [10].

Interleukin‐18 (IL‐18), a member of the IL‐1 cytokine family [11] and a member of the IL‐1 cytokine family [12], also drives proinflammatory signaling and has been linked to adverse cardiovascular remodeling [11] and ventricular dysfunction [12]. IL‐18 binding protein (IL‐18BP) provides physiological inhibition of IL‐18 activity [13], yet its behavior in chronic HF is incompletely defined. Although individual studies have reported elevated IL‐17 and IL‐18 and dysregulated Th17/Treg profiles in HF compared with healthy controls [14–17], this evidence has not been synthesized in a systematic review or meta‐analysis. The clinical utility of these circulating mediators as biomarkers of chronic HF particularly in ischemic heart disease and idiopathic dilated cardiomyopathy remains uncertain.

Therefore, the objective of this study was to systematically evaluate IL‐17, IL‐18, Th17, and Treg levels in chronic HF to determine whether consistent abnormalities across studies support their involvement in HF pathogenesis and their potential as diagnostic or prognostic biomarkers.

## 2. Materials and Methods

### 2.1. This Review Was Directed Following the 2020 Preferred Reporting Items for Systematic Reviews and Meta‐Analyses (PRISMA) Guidelines

Electronic searches were conducted in MEDLINE and Embase with the following search strategy: ((“IL‐17”[Mesh]) or (“IL‐18” [Mesh] AND (“Heart Failure”[Mesh] OR “Heart Failure, Diastolic”[Mesh] OR “Heart Failure, Systolic”[Mesh]) from database inception to June 2024.

The inclusion criteria were primary research studies published in English that examined serum IL‐17 concentration in adults with HF or had HF as an outcome. The etiology of HF was restricted to HF due to ischemic (coronary) heart disease or HF of unknown etiology and to exclude myocarditis which is known to involve an inflammatory response. The exclusion criteria were individuals in the pediatric age group, non‐HF studies, abstracts, editorials, case reports, and reviews.

All references were uploaded to Covidence and were electronically merged to remove duplicates [18]. Two authors individually reviewed each study to determine their inclusion or exclusion (JS AND SS). The data extracted from each study were: study design, country in which the study was conducted, sample size, mean age, sex (% male), notable comorbidities, duration of follow‐up, study design, IL‐17 serum levels, type of HF, left ventricular ejection fraction, NYHA class, body mass index (BMI), HF incidence, survival data, and hazard ratios. Two reviewers (JS and SS) conducted data extraction, and a consensus was reached as any conflicts were resolved by agreement between reviewers.

### 2.2. Data Analysis

Forest plots were used to quantify results and depict the standard difference of means, 95% confidence interval (CI), and *p* value. Data analysis was conducted in comprehensive meta‐analysis and was assessed based on the magnitude of the point estimate and its associated precision. Heterogeneity was assessed through the *I* ^2^ statistics with a value above 50% being significant. Examination of potential publication bias was conducted using the classic fail‐safe N, using comprehensive meta‐analysis (Biostat Inc., NJ, USA).

## 3. Results

### 3.1. A Total of 1375 Studies Were Identified From Databases and Registers (Supporting Figure 1)

After removing 569 duplicates identified by Covidence, 806 studies remained for screening. One study was added through citation search. Following the screening process, 770 studies were excluded for not meeting the inclusion criteria, leaving 31 full texts. Fifteen of these had wrong outcomes, four were abstracts, three had wrong comparators, one was an animal study, and one had the wrong patient population.

The characteristics of the studies that examined IL‐17 serum levels in HF show a diverse range of studies (Table 1). Across all studies evaluating IL‐17 serum concentrations [15, 16, 19–23], the pooled weighted mean age of patients with HF was approximately 58.5 years compared to 54.1 years in control groups. The pooled proportion of males was also slightly higher among HF patients (58.6%) than controls (54.0%). These demographics are consistent with known epidemiology of HF and suggest reasonable comparability.

**TABLE 1:** Studies that examined L‐17 serum concentrations in heart failure.

Study	Country	Study design	Heart failure group			Control		
			*N*	Mean age	% male	*N*	Mean age	% male
Huang et al. 2009	China	Prospective cohort study	30	52.3 ± 6.2	63.30%	20	46 ± 4.2	55%
Yi et al. 2009	China	Prospective cohort study	60	51.3 ± 10.4	50%	60	73 ± 13	50%
Zhu et al. 2012	China	prospective cohort study	92	NR	63%	59	NR	55.90%
Li et al. 2016	China	Prospective cohort study	70	73.54 ± 11.5	57.10%	35	70.75 ± 14.08	54.30%
Cai et al. 2016	China	Prospective cohort study	150	67.82 ± 12.27	63%	50	NR	NR
Wang et al. 2017	China	Prospective cohort study	127	48.1 ± 4.5,	51.84%	407	32.0 ± 13.3	66.93%
Sun et al. 2019	China	Prospective cohort study	210	150	58%	60	50.12 ± 3.15	70%
Zhao et al. 2024	China	Retrospective cohort study	429	79	51.70%	NA	NA	NA
Baumhove et al. 2024	The Netherlands	Retrospective cohort study	2023	IL‐17D 1st Quartile: 62.8 IL‐17D 2nd Quartile: 67.3 IL‐17D 3rd Quartile: 70.8 IL‐17D 4th Quartile: 74.4	IL‐17D 1st Quartile: 77 IL‐17D 2nd Quartile: 88.2 IL‐17D 3rd Quartile: 89.7 IL‐17D 4th Quartile: 89.6	NA	NA	NA

*Note*: Median, 25^th^–75^th^ percentile. *N* = sample size. No control group was present in this study.

Abbreviations: NA, not applicable; NR, not reported.

**FIGURE 1:**
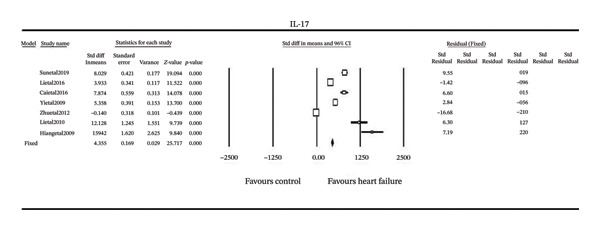
Forest plot of the meta‐analysis of all studies of serum IL‐17 in heart failure.

The weighted mean difference (WMD) of IL‐17 serum concentrations between individuals with HF and controls was examined across included studies (Figure 1). Patients with HF consistently show higher IL‐17 levels compared to control groups as indicated by positive mean differences in all but one study [20]. The pooled random‐effects estimate showed a significant overall increase in IL‐17(MD = 40.00 pg/mL, 95% CI [6.04, 73.96], *p* < 0.001). Heterogeneity was substantial (I^2^ = 100%). The study by Huang et al. (2009) reported largest effect size (MD = 135.44 pg/mL). Assessment of publication bias indicated strong result stability with more than 2800 hypothetical null‐effect studies required to eliminate statistical significance.

**FIGURE 2:**
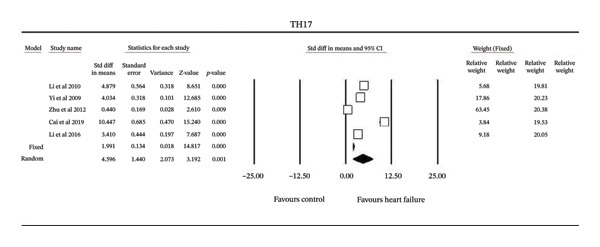
Forest plot of the meta‐analysis of all studies of serum TH‐17 in heart failure.

Serum Th17 cell levels were also compared between HF patients and controls (Figure 2). Across five studies, Th17 cell frequencies (Th17/CD4+ T‐cells) were consistently higher in HF. The pooled random‐effects analysis confirmed significantly elevated Th17 levels in HF (MD = 1.59%, 95% CI (0.88, 2.30]; *Z* = 3.19, *P* = 0.001). Heterogeneity among studies was high (*Q* = 314.4, I^2^ = 99%, Tau^2^ = 10.15 + 9.2). Robustness testing showed that 567 additional null‐effect studies would be needed to attenuate the observed significance.

**FIGURE 3:**
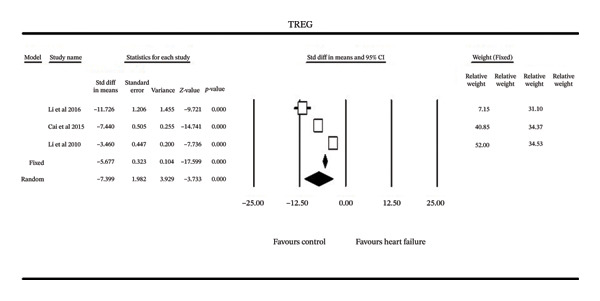
Forest plot of the meta‐analysis of all studies of serum TREG in heart failure.

T‐regulatory cell (Treg) concentrations were assessed in three studies (Figure 3). All demonstrated significantly reduced Treg levels in HF relative to controls. The pooled common effect model showed significant reduction (MD = 2.63%, 95% CI [−2.73, −2.53]), which remained significant under the random‐effects model (MD = −2.96%, 95% CI [4.52, −1.40]). Heterogeneity was substantial (I^2^ = 99%).

**FIGURE 4:**
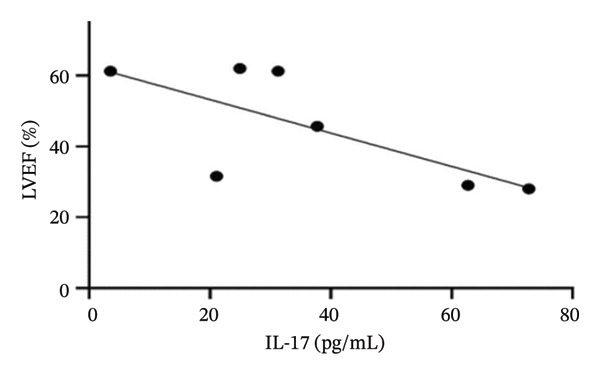
Serum IL‐17 and left ventricular ejection fraction in all studies that reported these data.

Several studies reported both IL‐17 serum concentration and LVEF in patients with chronic HF as well as controls (Figure 4). A negative correlation was observed between serum IL‐17 concentrations and LVEF across studies reporting both measures (*r* = −0.48), consistent with the hypothesis that elevated IL‐17 may be associated with impaired systolic function. However, this correlation did not reach statistical significance (*p* = 0.52), likely reflecting the limited number of available studies.

Five studies compared serum levels of IL‐18 in HF vs controls (Tables 2 and 3). Across these five studies [24–28] evaluating IL‐18 serum concentrations, the pooled weighted sample included 214 patients with HF and 144 controls, representing cohorts from Asia, Australia, and Europe. The weighted mean age among HF patients was 63.7 years compared with 57.9 years in control groups. The weighted proportion of males was 65.4% in HF cohorts and 57.6% in the control populations.

**TABLE 2:** Studies of prognosis according to serum concentrations of IL‐18.

Study	Country	Study design	Heart failure group			Control		
			Sample size	Mean Age	% Male	Sample size	Mean Age	% Male
Alfieri et al. (2008)	Italy	Retrospective cohort study	HF baseline = 38, HF + carvedilol = 22	HF baseline = 38, HF + carvedilol = 22	NR	40	NR	NR
Naito et al. (2002)	Japan	Prospective cohort study	34	NR	NR	10	NR	NR
Cheng et al. (2009)	China	RCT	CHFa = 37, CHFc = 35	CHFa = 61 ± 15, CHFc = 60 ± 10	CHFa = 59%, CHFc = 57%	20	60 ± 7	65%
Eslick et al. (2008)	Australia	Retrospective cohort study	30	62	70%	16	49	62.5%
Mallat et al. (2004)	France	Prospective cohort study	DCM = 13, ICM = 6	DCM = 53.1 ± 3.2, ICM = 53.5 ± 4.9	DCM = 76.9%, ICM = 100%	NA	NA	NA
Hartford et al. (2010)	Sweden	Prospective cohort study	306	69 ± 8	65%	656	61 ± 10	90%
Sanchez et al. (2014)	Spain	Retrospective cohort study	124	83 ± 5	32.20%	NA	NA	NA
Bouwens et al. (2020)	The Netherlands	Prospective cohort study	250	68 (58–76)^∗^	74%	NA	NA	NA
Jia et al. (2023)	USA	Prospective cohort study	5672	75.4 ± 5.1	42%	NA	NA	NA

*Note: N* = sample size. No control group was present in this study.

Abbreviations: CHF, congestive heart failure; DCM, idiopathic dilated cardiomyopathy; ICM, ischemic dilated cardiomyopathy; NA, not applicable; NR, not reported; RCT, randomized controlled trial.

^∗^Median, 25^th^–75^th^ percentile.

**TABLE 3:** Comorbidities in studies examining the prognostic role of interleukins in heart failure.

Study	Presence of CAD	Presence of diabetes mellitus	Presence of atrial fibrillation
Huang et al. 2009	NR	NR	NR
Yi et al. 2009	NR	NR	NR
Zhu et al. 2012	NR	NR	NR
Li et al. 2016	NR	NR	NR
Cai et al. 2016	NR	NR	NR
Sun et al. 2019	NR	NR	NR
Zhao et al. 2024	245 (57.1%)	148 (34.5%)	154 (35.9%)
Baumhove et al. 2024	NR	IL‐17D 1st Quartile: 143 (28.1 IL‐17D 2nd Quartile: 169 (33.3 IL‐17D 3rd Quartile: 169 (33.3%) IL‐17D 4th Quartile: 164 (32.3%)	IL‐17D 1st Quartile: 178 (35.0%) IL‐17D 2nd Quartile: 205 (40.4%) IL‐17D 3rd Quartile: 251 (49.4%) IL‐17D 4th Quartile: 291 (57.3%)
Hartford et al. (2010)	NR	NR	NR
Sanchez et al. (2014)	NR	NR	NR
Bouwens et al. (2020)	119 (48%)	77 (31%)	97 (39%)
Jia et al. (2023)	NR	1826 (32.3%)	NR

Abbreviations: CAD, coronary artery disease; CHF, congestive heart failure; CV death, cardiovascular death; MI, myocardial infarction; non‐CV death, noncardiovascular death; NR, not reported.

**FIGURE 5:**
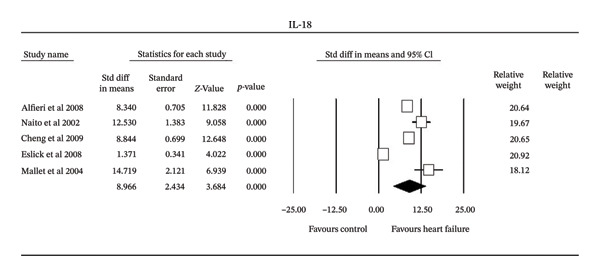
Forest plot of the meta‐analysis of all studies of serum IL‐18 in heart failure.

All five studies reported a statistically significant increase in IL‐18 levels in HF patients (Figure 5). The standard difference in the means showed that each study had a significantly higher IL‐18 in HF than controls. Mean differences ranged from 172.00 pg/mL [25] to 388.65 pg/mL [24]. The pooled mean difference was 251.59 pg/mL (95% CI: 177.24–325.93 pg/mL) with a random effects model. There was a high heterogeneity (*Q* = 208.2, *I* ^2^ = 98.1, and Tau^2^ = 28.1 ± 26.7), which reflects considerable variability across studies although none were significant. Examination of potential publication bias found that it would require 511 additional studies to be added to the current data to yield a nonsignificant result.

## 4. Discussion

Our study demonstrates that serum IL‐17 concentrations are significantly elevated in individuals with HF compared to healthy controls and that higher IL‐17 levels correlate with greater disease severity, including higher NYHA class and lower LVEF. We also found that circulating Th17 cells are increased and T‐regulatory cells (Tregs) are reduced in HF, supporting a shift toward proinflammatory adaptive immune activation. To our knowledge, this is the first meta‐analysis to concurrently evaluate IL‐17, IL‐18, Th17, and Treg alterations in chronic HF.

Il‐17 is a proinflammatory cytokine primarily produced by Th17 cells and stimulates downstream release of IL‐1β, IL‐6, and TNF‐alpha. Preclinical studies show that IL‐17 contributes to myocardial fibrosis, stiffness, and ventricular remodeling. Zhou et al. reported that IL‐17A exacerbates post‐myocardial infarction fibrosis, in part by increasing MMP‐2 activity [29], while Zan et al. linked higher IL‐17 with increased extracellular matrix deposition [30], suggesting relevance to HFpEF. IL‐17 also promotes cardiomyocyte apoptosis through both intrinsic apoptotic signaling and mitochondrial pathways [29] and may impair contractility by disrupting Cav1.2‐ and SERCA‐mediated calcium handling [8]. Collectively, these mechanisms support a biological link to both HFrEF (heart failure with reduced ejection fraction) and HFpEF (heart failure with preserved (normal) ejection fraction).

The reduction in circulating Tregs observed across included studies may represent a key mechanism driving Th17 expansion and elevated IL‐17 levels in HF [9, 16, 22, 23]. This imbalance between regulatory and proinflammatory T‐cell subsets highlights a central role for adaptive immunity in HF progression and may offer opportunities for immune‐targeted therapies. The established utility of biomarkers in HF risk stratification [31] further suggests IL‐17 may have clinical value as an inflammatory biomarker in both disease monitoring and prognostication.

Prior cytokine‐targeted strategies have shown mixed results in HF. While TNF‐alpha inhibition with Etanercept failed to improve outcomes [32], infliximab demonstrated some favorable effects [33]. Whether IL‐17 blockade or other modulations of T‐cell responses could yield therapeutic benefit remains an important direction for future investigation.

Il‐18, another proinflammatory IL‐1 family cytokine, was also significantly elevated in HF across included studies. Mechanistically, IL‐18 induces Th1‐type responses, activates macrophages, and stimulates cytokines including TNF‐alpha and IL‐6 [11]. In murine models, IL‐18 triggers myocardial fibrosis [34] and hypertrophy [35] through JNK/Akt signaling. Human data mirror these findings, with higher IL‐18 associated with more advanced NYHA functional class and lower LVEF [36] reinforcing its potential as a severity marker.

Data on whether IL‐17 or IL‐18 relates differentially to ischemic versus nonischemic etiologies remain inconsistent [17, 22, 37], Similarly, some evidence suggests distinct associations between IL‐17 subtypes (e.G., IL‐17D vs IL‐17A) and HFpEF vs HFrEF [33], emphasizing the need to investigate cytokine isoforms separately.

This review has several strengths, including synthesis of molecular, mechanistic, and clinical data to characterize the IL‐17/Th17/Treg axis and IL‐18 in HF. However, limitations exist. Most studies did not stratify HF based on EF phenotype or etiology, and a few differentiated IL‐17 subtypes (A‐F), limiting mechanistic specificity. Comorbidities such as diabetes are known to elevate IL‐17 levels, thereby resulting in a confounding factor [38]. Sex imbalance was also present across studies, with higher male participants. Emerging evidence suggests potential sex‐specific differences in IL‐17 signaling, introducing another source of confounding. Data on whether IL‐17 or IL‐18 relates differentially to ischemic versus nonischemic etiologies remain inconsistent [1. However, limitations exist. Most studies did not stratify HF based on EF phenotype or etiology, and few differentiated IL‐17 subtypes (A–F), limiting mechanistic specificity. Comorbidities such as diabetes are known to elevate IL‐17 levels [51] and thus may introduce confounding. Sex imbalance was present across studies, with higher male representation; emerging data suggest potential sex‐specific IL‐17 signaling differences [15, 39], warranting further research. Additionally, most included studies were conducted in China, raising concerns regarding geographic generalizability [6, 27, 49]. Similarly, some evidence suggests distinct associations between IL‐17 subtypes (e.g., IL‐17D vs. IL‐17A) and HFpEF vs. HFrEF [33], emphasizing the need to investigate cytokine isoforms separately.

This review has several strengths, including synthesis of molecular, mechanistic, and clinical data to characterize the IL‐17/Th17/Treg axis and IL‐18 in HF. Most studies did not stratify HF based on EF phenotype or etiology and few differentiated IL‐17 subtypes (A–F), limiting mechanistic specificity. Comorbidities such as diabetes known to elevate IL‐17 levels and emerging sex‐specific IL‐17 signaling differences also need to be further explored. Lastly, most studies were conducted in China, raising concerns regarding geographic generalizability.

Our study found that serum concentrations of IL‐17 are significantly higher in persons with HF compared to individuals without HF. In addition, higher serum levels predict a greater likelihood of major adverse cardiovascular events in patients with chronic HF. In addition, Th17 cells are elevated in patients with chronic HF, while Tregs are downregulated. To our knowledge, there was no prior meta‐analysis on IL‐17, Th17, and Tregs within the context of chronic HF. We propose that IL‐17 is intimately linked with chronic HF and represents a mechanism by which dysfunctional adaptive immunity can have nefarious effects upon the myocardium. Our synthesis of the data points to the possibility of a novel therapeutic approach to target Th17‐ and IL‐17‐mediated inflammation in patients with HF.

**FIGURE 6:**
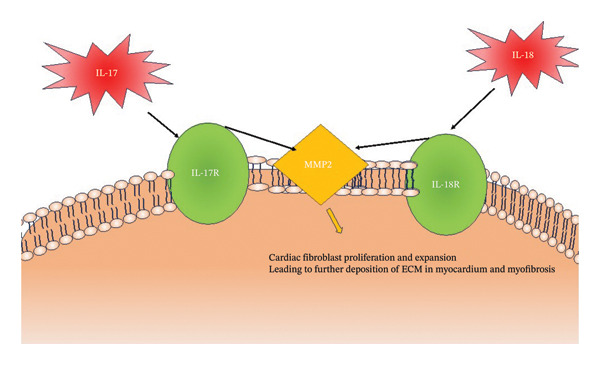
The molecular pathways by which IL‐17 and IL‐18 lead to cardiac fibrosis.

**FIGURE 7:**
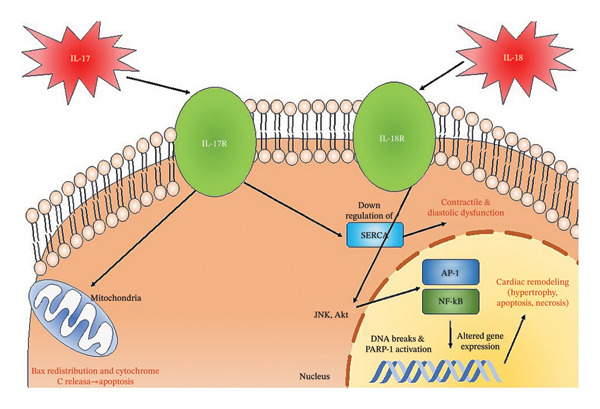
The molecular pathways by which IL‐17 and IL‐18 lead to contractile dysfunction.

IL‐17 is a proinflammatory cytokine primarily produced by T‐lymphocytes and induces production of other inflammatory cytokines as well as IL‐1B, IL‐6, TNF‐a, and IL‐1B. Animal studies have elucidated the role of IL‐17 in initiating myocardial fibrosis [29]. Zhou et al. found that IL‐17A plays a critical role in left‐ventricular remodeling following acute myocardial infarction [29]. IL‐17A exacerbates fibrosis by increasing Metalloproteinases‐2 (MMP2) activity (Figure 6). IL‐17 has also been attributed to increased myocardial stiffness with increased fibrosis in the heart. Zan et al. found that higher levels of IL‐17 are associated with increased amounts of extracellular matrix fibrosis supporting the postulate that IL‐17 may play a role in myocardial fibrosis [30]. This suggests a role in HfpEF. IL‐17A can play a role in inducing cardiomyocyte apoptosis through multiple mechanisms, including the intrinsic pathway, p53 phosphorylation, Bax redistribution, and cytochrome C release [29]. Xue et al. postulated that IL‐17 promotes the development of HF through interfering with calcium handling within cardiomyocytes [8]. They found that cardiomyocytes from IL‐17 knockout mice had greater expression of CaV1.2 and SERCA and had superior calcium handling and contraction, indicating a role for IL‐17 to disturb cardiomyocyte calcium handling and ventricular contraction through CaV1.2 and SERCA [8] (Figure 7).

Our review summarizes the complex interplay between downregulation of Tregs and the subsequent increase of Th17 cells and IL‐17 in chronic HF and reveals a crucial component of T‐cell‐mediated adaptive innate immunity in chronic HF. The data suggest that Th17 cells [22] are elevated in patients with chronic HF, while Tregs are downregulated [9, 16, 22, 23]. Downregulation of Tregs may lead to a dysfunctional increase in Th17 activity, which subsequently increases serum IL‐17 concentrations in HF. IL‐17 plays a role in disturbing calcium handling [8], thereby potentially playing a role in HFrEF [40]. It also causes cardiac fibroblast proliferation [29] that could lead to myocardial stiffness of HFpEF [41] (Figure 7).

We concluded that serum IL‐17 concentrations are associated with greater degrees of HF severity. There is a positive correlation between NYHA class and IL‐17 serum concentration and the number of Th17 in serum [15, 16, 21, 22]. This supports the role of IL‐17 and inflammation as a possible causative factor of HF because the greater the elevation of serum IL‐17, the greater the impairment in HF. The utility of biomarkers in HF [31] suggests the potential of IL‐17 as a biomarker for HF.

Prior clinical trials have also explored the impact of blocking cytokines resulting from IL‐17 activation. Earlier trials blocking TNF‐α through Etanercept did not find significant benefits in chronic‐HF [32]. However, a recent trial exploring the blockade of TNF‐α through infliximab for HF proved to have positive results [33]. However, more research is needed to determine whether cytokine blockade is a viable therapeutic strategy.

Downregulation of Tregs is also present in HF. Li et al. found that Tregs were decreased in patients with DCM, while the ratio Tregs/Th17 and serum IL‐17 levels were significantly higher [23]. This suggests that downregulation of Tregs might be a mechanism by which IL‐17 is elevated in patients with cardiomyopathy. Other studies have also found that elevated Treg serum concentrations are associated with better HF outcomes [42].

Data regarding the association of IL‐17 with the specific etiology of HF are conflicting, with some studies suggesting a stronger relationship in HF due to ischemic heart disease [22], while others do not [43]. The type of HF may influence the relationship between cytokine levels and HF. HF with preserved ejection fraction is more likely to be associated with inflammation [44–46]. A retrospective cohort study by Baumhove et al. indicated that higher serum concentrations of IL‐17D were associated with HFpEF as opposed to HFrEF [33]. This did conflict with several other studies in our review which had indicated that IL‐17 serum concentrations were directly associated with NYHA class and LVEF. This contrast may be explained by the possibility that IL‐17D and other subtypes of IL‐17 may have different effects on the heart.

IL‐18, a member of the IL‐1 family, is a proinflammatory cytokine with multiple biologic functions [11] In concert with IL‐12, IL‐18 stimulates Th1‐mediated immune responses; by itself, IL‐18 can stimulate Th2 cytokine production IL‐18, originally named as an interferon *γ* inducing factor (IGIF), and can induce TNFα and IL‐6 in murine macrophages [11]. IL‐18 in mice upregulates myocardial JNK and Akt signaling pathways, leading to an increase in cardiac fibroblast migration and proliferation with resulting myocardial fibrosis [34]. Elevated levels of IL‐18 play an important role in cardiomyocyte hypertrophy [35]. This also correlates with human studies. Ling et al. found a positive correlation between higher NYHA classifications and IL‐18 serum concentrations. Eslick et al. also supported this observation by finding that IL‐18 serum concentrations had a negative correlation with LVEF%, i.e., higher IL‐18 levels were associated with lower left ventricular ejection fraction [36]. Thus, IL‐18 serum concentration could be a predictor of severity of both HF and LVEF.

Data regarding whether IL‐18 could predict the etiology of HF is unresolved. Yamaoko‐Tojo et al. found that patients with dilated cardiomyopathy secondary to ischemic heart diseases had significantly higher serum concentrations of IL‐18 compared to patients with HF of nonischemic etiology [37]. In contrast, Ji et al. found no differences between IL‐18 and the etiology of CHF [17]. IL‐18 is linked to hypertension which is a well accepted cause of heart failure [47]. Further research is needed to distinguish whether IL‐18 serum concentration can be used to differentiate between HFpEF and HFrEF.

Our review presents several strengths. Most studies had a long‐term follow‐up, as all but two studies were tracking patients for a duration of two or more years. Nevertheless, further studies elucidating sex‐based differences of IL‐17 serum concentrations in patients with HF and studies focusing on differentiating IL‐17 subtypes within chronic HF are needed, and further studies with larger sample sizes are also needed to elucidate the specific roles and differences in IL‐17 and IL‐18 concentrations among CHF of different etiologies and whether there are differences in HFpEF and HFrEF.

However, there are several limitations of the study that warrant discussion. Meta‐analysis depends on the rigor of each study. Many of the papers did not stratify the type of HF or the ejection fraction. Furthermore, many studies also did not characterize the subtype of interleukin IL‐17, from A to F, and many studies focus on either IL‐17D or IL‐17A. Further studies are needed to elucidate whether the subtypes of IL‐17 differ in their impact on the heart, and future studies should also elucidate the specific subtype of IL‐17 that is elevated in patients with HF. Another limitation was the differences between studies in the frequencies of comorbidities. For instance, some patients had diabetes and hypertension [48], which may be associated with inflammation, as patients with type‐2 diabetes mellitus have higher serum concentrations of IL‐17 [38]. This may represent a confounding variable. Another consideration is the sex distribution of the study participants. The samples exhibited a higher representation of males compared to females. Yi et al. found significantly higher IL‐17 serum concentrations in men compared to women [15]. Interestingly, this may correlate with studies that indicate men have a higher rate of death from congestive HF [15]. In contrast, from a genomic standpoint, Raafs et al. found that men with chronic HF had significantly lower IL‐17 and Th17 expression [39]. This conflicting finding indicates that further studies are needed to explore the sex‐based differences in Th17 and IL‐17 mRNA expression and protein serum concentration. Another limitation was that majority of our studies were from China raising the possibility that there might be geographic limitations and confounding factors that may influence the results as well.

## 5. Conclusion

In conclusion, both IL‐17 and IL‐18 appear to play a role in HF. Further research is needed to elucidate whether the etiology and type of HF can be distinguished by serum concentrations of IL‐17 or IL‐18. Additionally, studies are also needed to examine the different subtypes of IL‐17 cytokines as well as to elucidate whether they have a differential role in the pathogenesis of chronic HF.

**Nomenclature**
IL‐17: Interleukin‐17
IL‐18: Interleukin‐18
Tregs: Regulatory T cells
Th17 cells: T helper 17 cells
HFpEF: Heart failure with reduced ejection fraction
HFrEF: Heart failure with preserved (normal) ejection fraction
BNP: Brain natriuretic peptide
NT‐proBNP: N‐terminal pro B‐type natriuretic peptide

## Author Contributions

Sohat Sharma: data extraction, analysis, and manuscript writing.

Jayant Seth and Cameron J. Leong: data extraction and manuscript writing.

Simon W. Rabkin: conceptualization, supervision, and manuscript writing.

## Funding

There was no funding support for this study.

## Disclosure

The manuscript has been previously presented as abstract presented at a scientific meeting https://onlinecjc.ca/article/S0828-282X(25)00906-7/abstract.

## Conflicts of Interest

The authors declare no conflicts of interest.

## Supporting Information

Additional supporting information can be found online in the Supporting Information section.

## Supporting information


**Supporting Information** Supporting Figure 1. PRISMA flow diagram.

## Data Availability

Data sharing is not applicable to this article as no datasets were generated or analyzed during the current study.
