# Supplementary figures and images for: Interleukin‐17, Interleukin‐18, T Regulatory Cells, and T‐Helper 17 (Th17) Cells Play a Role in Heart Failure: A Systematic Review and Meta‐Analysis
Source: Cardiol Res Pract. 2026 May 24;2026:5979003. doi: 10.1155/crp/5979003 (PMC13199857; doi:10.1155/crp/5979003)

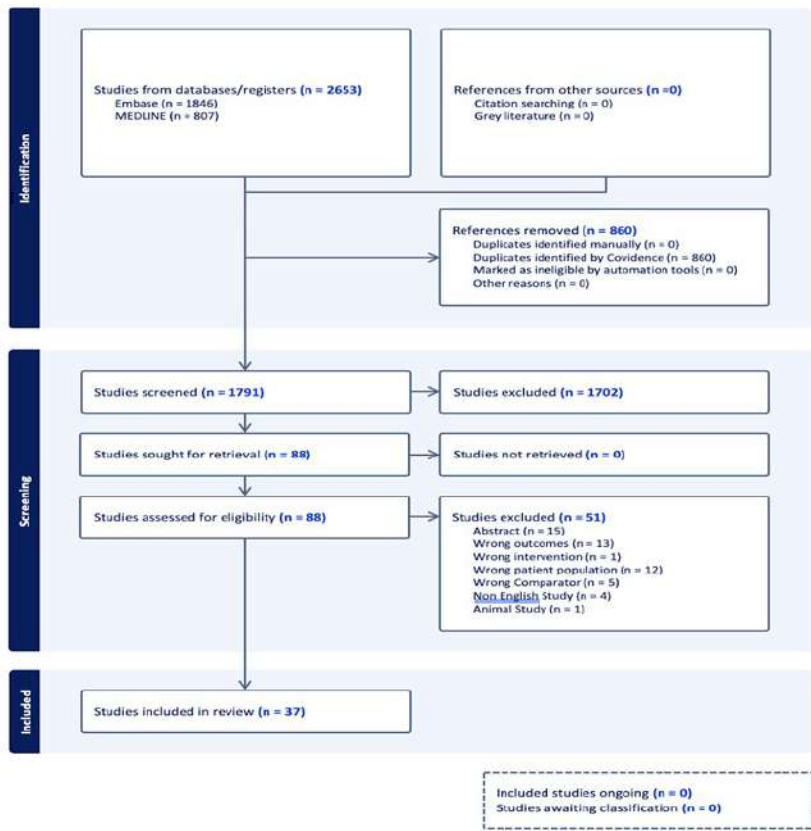

Supplement: Supplementary file 1 — Supporting Information Supporting Figure 1. PRISMA flow diagram. [file CRP-2026-5979003-s001.pdf]
